# ZRO Drift Reduction of MEMS Gyroscopes via Internal and Packaging Stress Release

**DOI:** 10.3390/mi12111329

**Published:** 2021-10-29

**Authors:** Pengfei Xu, Zhenyu Wei, Lu Jia, Yongmei Zhao, Guowei Han, Chaowei Si, Jin Ning, Fuhua Yang

**Affiliations:** 1 Engineering Research Center for Semiconductor Integrated Technology, Institute of Semiconductors, Chinese Academy of Sciences, Beijing 100083, China; xupengfei@semi.ac.cn (P.X.); zywei97@semi.ac.cn (Z.W.); jialu@semi.ac.cn (L.J.); ymzhao@semi.ac.cn (Y.Z.); ningjin@semi.ac.cn (J.N.); 2 College of Materials Science and Opto-Electronic Technology, University of Chinese Academy of Sciences, Beijing 100049, China; 3 School of Electronic, Electrical and Communication Engineering, University of Chinese Academy of Sciences, Beijing 100049, China; 4 State Key Laboratory of Transducer Technology, Chinese Academy of Sciences, Beijing 100083, China

**Keywords:** ZRO drift, MEMS gyroscope, internal and packaging stresses, finite element analysis, stress release

## Abstract

Zero-rate output (ZRO) drift induces deteriorated micro-electromechanical system (MEMS) gyroscope performances, severely limiting its practical applications. Hence, it is vital to explore an effective method toward ZRO drift reduction. In this work, we conduct an elaborate investigation on the impacts of the internal and packaging stresses on the ZRO drift at the thermal start-up stage and propose a temperature-induced stress release method to reduce the duration and magnitude of ZRO drift. Self-developed high-Q dual-mass tuning fork gyroscopes (TFGs) are adopted to study the correlations between temperature, frequency, and ZRO drift. Furthermore, a rigorous finite element simulation model is built based on the actual device and packaging structure, revealing the temperature and stresses distribution inside TFGs. Meanwhile, the relationship between temperature and stresses are deeply explored. Moreover, we introduce a temperature-induced stress release process to generate thermal stresses and reduce the temperature-related device sensitivity. By this way, the ZRO drift duration is drastically reduced from ~2000 s to ~890 s, and the drift magnitude decreases from ~0.4 °/s to ~0.23 °/s. The optimized device achieves a small bias instability (BI) of 7.903 °/h and a low angle random walk (ARW) of 0.792 °/√ h, and its long-term bias performance is significantly improved.

## 1. Introduction

Micro-electromechanical system (MEMS) gyroscopes, a kind of Coriolis effect-based inertial sensors, are widely employed in the fields of aerospace, positioning, navigation, and consumer electronics [1,2]. With the continuously developing design, manufacture, and control techniques of MEMS gyroscopes, their accuracy and precision have gained constant improvement [3,4]. Nowadays, a majority of MEMS gyroscopes work utilizing capacitive detection, mode-split, and phase-sensitive demodulation architecture. Nevertheless, this architecture will undoubtedly introduce a zero-rate output (ZRO) drift effect, severely limiting the gyroscope accuracy [5,6].

Many studies have reported the origin of gyroscope ZRO drift. For one thing, the gyroscope’s resonant frequency is susceptive to the Joule heat generated inside the device when it is in operation [7]. Meanwhile, the outside environmental instability, such as packaging stress, temperature variations, and shock environments [8,9], is another main cause of its frequency drift. On this occasion, well-designed materials and structures with opposite temperature-related characteristics are often used for temperature compensation in practical gyroscope manufacturing [10,11], but these methods are inapplicable to most gyroscopes. For another, the drive frequency (f_drive_) can be used as a temperature indicator for the first-order temperature compensation, and thus a sub-degree-per-hour bias stability (1σ) is attained [12]. Nevertheless, this approach is ineffective due to the limitation of frequency measurement accuracy in some situations. The inevitable phase delay through the sense model induces partial quadrature fluctuations into the in-phase channel, and this in-phase and quadrature (IQ) coupling will exacerbate the ZRO drift [13]. A one-time phase self-compensation method was reported to eliminate the circuit phase delay, and the temperature sensitivity of ZRO drift was greatly reduced [14]. However, this method cannot always accurately reflect the true circuit phase delay. Qian Shen et al. investigated the bias-temperature relationship during the thermal start-up process and found that the gyroscope frequency variation is closely related to the heating of the gyroscope and its control circuit operation [15]. No efficient improvements are reported towards this puzzle. Moreover, the adhesion layer between the gyroscope and packaging shell may induce packaging stress when there appears environmental vibration or temperature instability [16,17]. However, the stress-induced changes on gyroscopes’ performances have not been thoroughly investigated. Gyroscopes’ internal stresses and the packaging stresses may also contribute to the ZRO drift properties. Nevertheless, no related research work has been reported. It is meaningful to expound on the effects of internal and packaging stresses on ZRO drift, and an effective approach is also in great need to reduce the duration and magnitude of ZRO drift.

In this work, we, for the first time, detailly investigate the impacts of the internal and packaging stresses on gyroscopes’ ZRO drift and demonstrate a temperature-induced stress release method to reduce the duration and magnitude of ZRO drift. We adopt self-developed high-Q dual-mass tuning fork gyroscopes (TFGs) with a dedicated control circuit to explore the correlations between ZRO drift and temperature at the thermal start-up stage. To uncover the gyroscope’s inside temperature distribution and the temperature-dependent stress variation in its working process, we built a rigorous finite element simulation model based on the actual device and package structure. Moreover, we propose a feasible method for system stress release by cyclically applying high and low temperatures onto devices. With the assistance of generated thermal stresses, the temperature sensitivity of internal and packaging stresses is significantly reduced, effectively reducing the impacts of temperature variation on the ZRO drift during TFGs’ thermal start-up processes. Notably, we conducted an in-depth study about the effects of the internal and packaging stresses on the ZRO drift and reported a genuine improvement toward the duration and magnitude of ZRO drift, providing a new promise for improving the long-term bias performances of MEMS gyroscopes.

The rest of this paper is organized as follows. Section 2 gives the device architecture and packaging structure of TFGs. Section 3 describes the research on the ZRO drift of TFGs. Moreover, the numerical simulation of internal and packaging stresses is given in Section 4. Section 5 presents the temperature-induced stress release process and its impact on the ZRO drift. Finally, Section 6 concludes this paper.

## 2. Device Architecture and Packaging Structure of TFGs

To investigate the impacts of gyroscopes’ internal stresses and the packaging stresses on ZRO drift at the thermal start-up stage, self-developed high-Q dual-mass TFGs [18], fabricated with through-silicon-via (TSV) and glass-in-silicon (GIS) reflow techniques [19], are adopted in this work.

**Figure 1:**
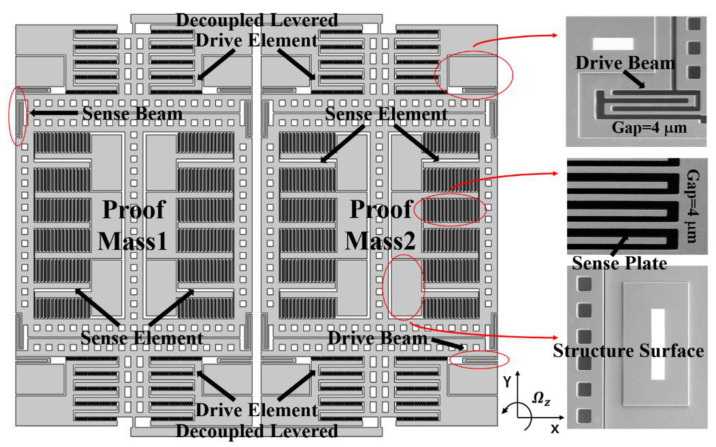
Typical schematic and SEM images of the as-fabricated dual-mass TFGs.

The schematic and scanning electron microscope (SEM) images of the TFGs are shown in Figure 1. A typical TFG consists of two symmetrically-decoupled proof masses and two symmetrically-decoupled levers, ensuring the suppression of common modes and the parasitical low-frequency in-phase mode. When the electrostatic excitation is applied on the drive elements, the proof masses vibrate along the drive axis at the initial stage. Subsequently, they vibrate with a constant amplitude and also a constant frequency under the negative feedback control. Based on the Coriolis effect [20], a corresponding output, which is proportional to the input angular velocity, will be obtained through the sense elements when there is an external angular velocity input at *Z*-axis. As revealed by the SEM image, TFGs are with a flat surface, distinct comb teethes, and smooth sidewalls, attesting our mature device fabrication process.

**Figure 2:**
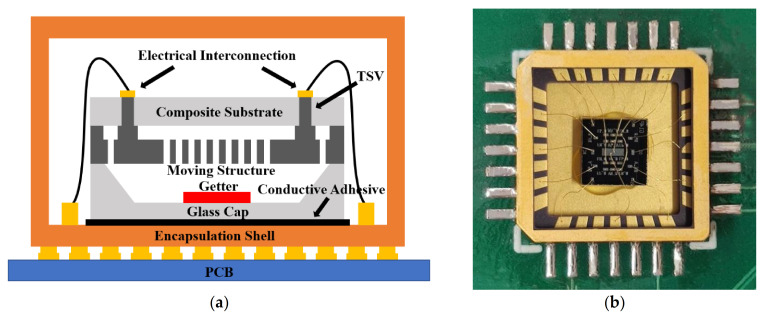
(**a**) Schematic illustration of the TFG architectures and (**b**) a photograph for the ceramic carrier of the vacuum-packaged MEMS TFGs.

As illustrated in Figure 2a, the dual-mass TFG is composed of a composite substrate, a moving structure, and a glass cap. The glass cap of the wafer-level packaged (WLP) TFG is affixed to the encapsulation shell using the conductive adhesive. The electrical interconnection between the aluminum electrodes and the shell electrodes is realized through wire bonding. Besides, the encapsulation shell and the control circuit system are integrated on a printed circuit board (PCB) to realize loop control and signal processing. To reduce the stress mismatch between the WLP TFG and the encapsulation shell, a ceramic carrier (CLCC28), whose thermal coefficient of expansion (TCE) is close to silicon, is used here, and its photograph is shown in Figure 2b.

**Figure 3:**
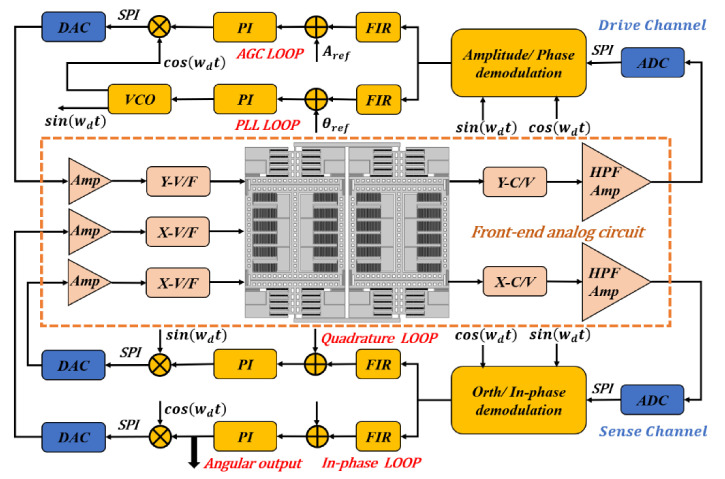
A block diagram of the control circuit system.

The block diagram of the control circuit system is exhibited in Figure 3, in which the front-end analog circuit, the signal conversion module, and the field-programmable gate array (FPGA) can be clearly recognized. The front-end analog circuit is mainly responsible for signal acquisition, capacitor-voltage conversion, signal amplification, and filtering. The capacitance signals of TFGs are modulated and demodulated with ring diodes to improve the signal-to-noise ratio (SNR). A signal conversion module, which contains two 14-bit analog-to-digital converters (ADC, ADS5553) and three 14-bit digital-to-analog converters (DAC, DAC2904), is used for the conversions of analog and digital signals. Thereafter, the acquired signals transmit from the conversion module to the FPGA via the serial peripheral interface (SPI) protocol. Moreover, signal processing and loop control are implemented in the FPGA, in which the automatic gain control (AGC) and phase-locked loops (PLLs) are designed in the drive mode to stabilize the vibration amplitude and frequency. Both the quadrature error correction and the in-phase loop are fully loop-locked to suppress the quadrature error and read the angular velocity signal. Hence, the sense mode of TFGs is rebalanced by using electrostatic forces. In the following sections, all tests are performed using the same control circuit system described in Figure 3 to ensure consistent experimental conditions.

**Figure 4:**
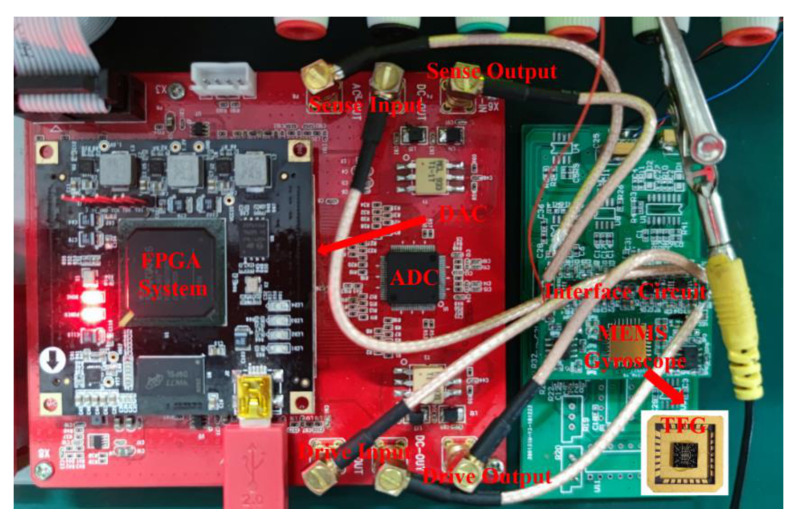
A photograph for the constructed testing system.

A typical photograph of the constructed experimental platform is shown in Figure 4. The control loops are embedded in FPGA, and the ceramic carrier is mounted on the interface circuit. As shown in the photograph, this platform is composed of three parts, and a computer is used for data transmission with FPGA through a universal synchronous asynchronous receiver transmitter (USART).

**Figure 5:**
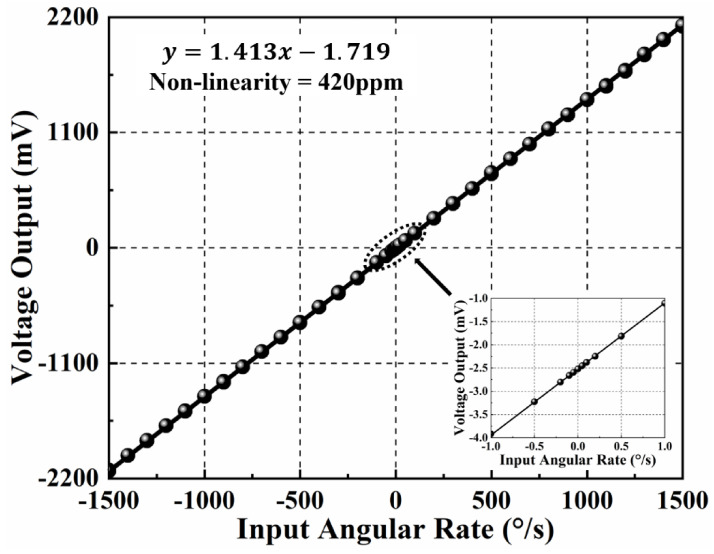
As-measured angular rate response and the non-linearity characteristics of MEMS TFGs.

The TFG and control circuit system is placed on a single-axis rate table (TBL-S1101-AT03) to investigate their responses to the input angular velocity. Firstly, the control circuit system is powered on, and the angular velocity experiment is conducted after the TFG works stably. When an input angular rate varying from −1500 °/s to +1500 °/s is applied, the output angular rate signal is collected for 2 min, and the average measured value is taken as the standard value of the sample. As-measured angular rate response of MEMS TFGs and their non-linearity characteristics are shown in Figure 5, where the measuring range is over 1500 °/s and the resolution is 0.1 °/s. Besides, the scale factor (SF) and non-linearity are 1.413 mV/°/s and 420 ppm, respectively. The experimental results indicate that the TFGs are with a high-resolution, wide-range, and highly-linear angular velocity response. It is proved that the designed TFGs have excellent device characteristics, and the control loop can effectively make proof masses work at the equilibrium position through the loop-locked force feedback. Hence, it is feasible to explore the ZRO drift at the thermal start-up stage using these high-Q dual-mass TFGs.

## 3. Research on the ZRO Drift of TFGs

**Figure 6:**
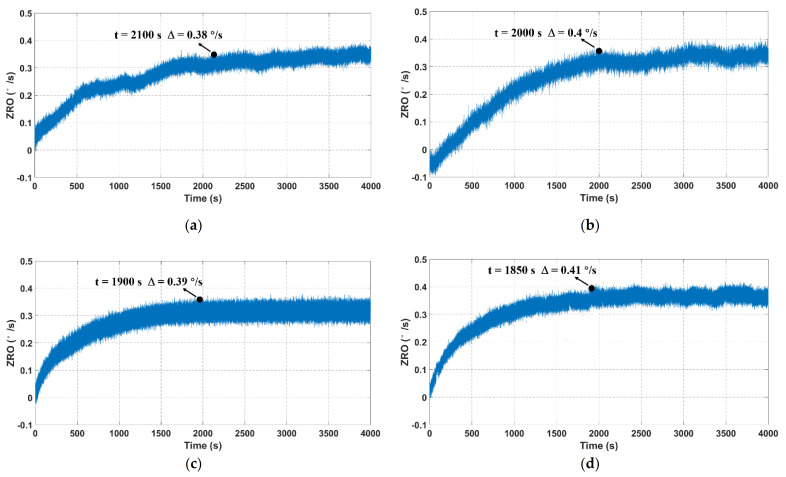
ZRO result comparisons between four TFGs at a sampling rate of 1 kHz. (**a**–**d**) represent the ZRO of samples 1–4, respectively.

ZRO is one of the most important performance indicators of MEMS gyroscopes, and its inevitable drift severely limits the gyroscope precision and accuracy. Hence, it is meaningful to explore an effective method for the ZRO drift reduction. To deeply investigate the output characteristics of TFGs, four samples, all of which have similar drive and sense frequencies, are adopted to ensure the testing accuracy and reliability in this work. For ZRO measurement, the output angular rate is recorded at a sampling rate of 1 kHz for 4000 s at room temperature. The ZRO comparisons between four tested samples are illustrated in Figure 6. It can be seen that their ZRO results show an obvious drifting tendency with the operating time. Besides, the ZRO drifts with a gradually slow velocity and eventually reaches a relatively stable state after a certain time. The ZRO drift duration is ~2000 s, and its magnitude is approximately 0.4 °/s for each tested sample, as listed in Table 1. This large ZRO drift may lead to the reduction of phase-sensitive demodulation accuracy, and it also has serious impacts on the gyroscope performances, including bias instability (BI) and angle random walk (ARW). Hence, it is essential to investigate the origin of ZRO drift, uncover the drift mechanisms and explore an effective method to relieve the ZRO drift.

**Figure 7:**
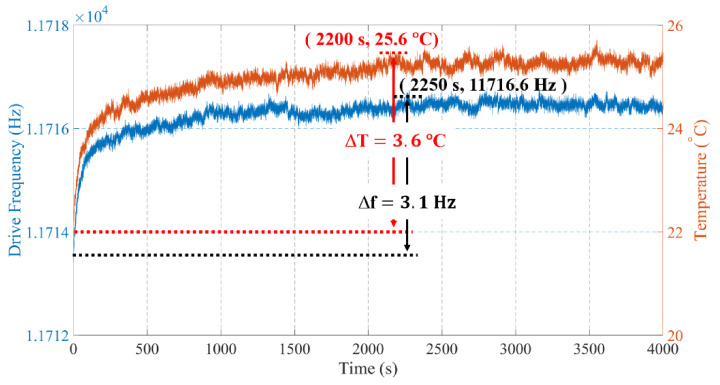
Measured device temperature and f_drive_ of sample1.

Many studies have discussed the mechanisms of ZRO drift in the past decades, and it is believed that the ZRO drift of gyroscopes mainly results from their inherent sensitivity to temperature variations [21,22]. To investigate the correlations between temperature and the ZRO drift, elaborate measurements of the device temperature and f_drive_ at the thermal start-up stage are conducted. As shown in Figure 7, the device temperature of TFGs rises rapidly within an initial 300 s since the power is on, and then the rising rate gradually slows down. The device temperature reaches a peak value of 25.6 °C after ~2200 s and then stabilizes, suggesting a temperature increase of ~3.6 °C. Besides, the f_drive_ has a similar variation tendency. It tends to be stable after ~2250 s, and the variation of f_drive_ is ~3.1 Hz. The testing results indicate that the ZRO drift has a strong correction with the f_drive_ and device temperature.

For one thing, the device parameters, such as Young’s modulus, are sensitive to temperature, causing the TFG frequency change with temperature variation [12]. Therefore, the f_drive_ is often used as one of the compensation indicators of the ZRO drift of gyroscopes. For another, the Joule heat generated by gyroscopes and interface circuits will change the device temperature during its working process. Besides, the TCE mismatch between the gyroscope and its package material leads to packaging stress. It is noted that this TCE mismatch also exists inside the gyroscope, causing significant impacts on the ZRO drift of gyroscopes.

It is essential to uncover the gyroscope’s inside temperature distribution and the temperature-dependent stress variation during its normal operation. However, the accurate correlation between stresses and temperature is not easy to obtain due to the complex TFG and package structure. Finite element simulation is a common method for analyzing certain specific situations. Therefore, a rigorous finite element simulation model, based on the actual device and package structure (shown in Section 2), is used to verify the existence of internal and packaging stresses and to investigate the temperature distribution and the relationship between temperature and stresses.

## 4. Numerical Simulation of Internal and Packaging Stresses

To investigate the impacts of internal and packaging stresses on the ZRO drift of MEMS gyroscope, we modeled the packaged TFGs with COMSOL Multiphysics software based on our practical devices. As illustrated in Figure 4, the PCB was designed to provide sufficient space between the gyroscope and the circuit components, avoiding the adverse effects of heat and thermal stress on the TFG performances when the inside components of the control circuit are working. Besides, the FPGA module, which generates a major proportion of heat, is designed on an additional layer of PCB. Therefore, we can effectively exclude the influences of the circuit component heating on the gyroscope performances. On this occasion, only the gyroscope itself and the package will be considered in the following simulation analyses.

**Figure 8:**
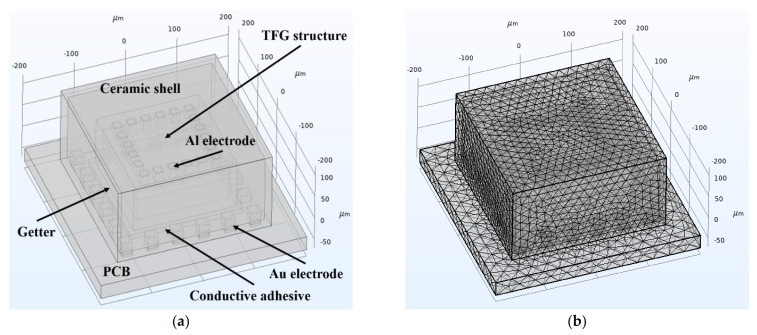
(**a**) Simulation model and (**b**) grid model of our packaged TFGs.

As shown in Figure 8a, a rigorous finite element simulation model is established based on the actual device and package structures of our fabricated devices. This model consists of a TFG structure, a glass cap, a ceramic shell, an electrode, the PCB, and other related parts. The solid mechanics and heat transfer in solid modules together with a thermal expansion multi-physics field are used to study the temperature and stress changes when TFGs are in a working process. The dimensions and essential material parameters of components in the simulation model are listed in Table 2, where the thermal conductivity κ means the ability of a material to conduct heat directly while the TCE represents the volume change for a unit temperature change. The grid model of the TFG structures and packaging system is shown in Figure 8b, in which the meshes are divided separately according to the structure and size of different regions to obtain more accurate simulation results.

**Figure 9:**
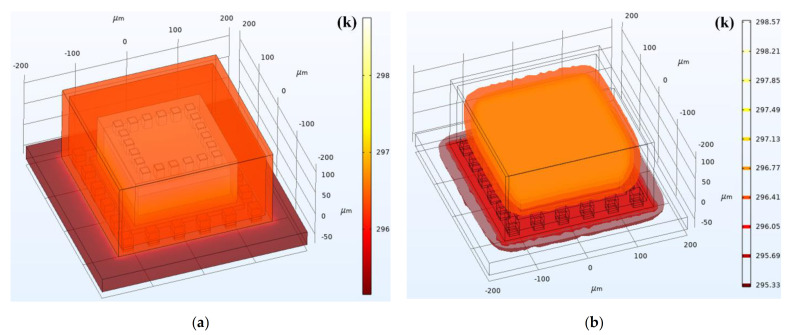
(**a**) Transient temperature distribution and (**b**) isothermals within TFGs during their working processes.

When setting the boundary conditions and initial conditions of the simulation model, the environment temperature was set as 295.15 K based on the actual initial device temperature. Since the influences of the circuit system were not considered, the TFG structure was set as the exclusive heat source in this simulation model. The transient temperature distribution of the TFG structures and packaging system is shown in Figure 9a. To maintain the ultra-high Q value of the TFG, the cavity was retained at a pressure of ~10^−5^ mbar in the manufacturing process. Hence, the heat conduction induced by the cavity air can be basically ignored. The heat generated by TFG is conducted to the ceramic shell through the conductive adhesive and metal electrodes, and eventually, most heat dissipates into the air. Besides, a portion of Joule heat was conducted to the top of the PCB through the contact between the ceramic shell bottom and PCB. Finally, there appears a balance between the heat generation in the working process of TFGs and the heat dissipation of the whole system, and the temperature, together with the f_drive_ of the TFG tends to be stable.

The isothermals within TFGs during their working processes are shown in Figure 9b, and it can be observed that the TFG maintains a relatively high temperature inside the device. Due to the mismatches resulting from materials or the process-induced residual stresses, the generated Joule heat may cause large strains inside the TFGs, which seriously affects the gyroscope performances and even causes the TFG failure. Therefore, it is essential to deeply investigate the stress distribution inside the device and that between the device and packaging structure during the working process of TFGs.

**Figure 10:**
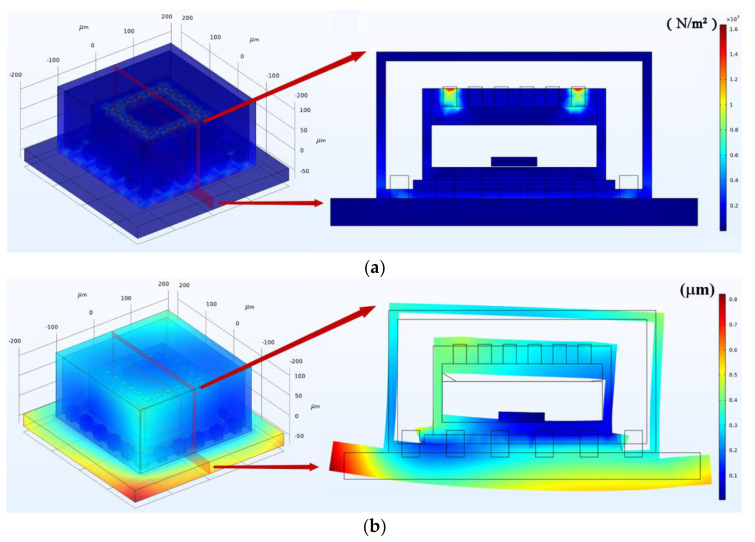
The (**a**) stress and (**b**) displacement distribution within TFGs at temperature equilibrium stage.

As depicted in Figure 10a, the heat-induced stress is ~10 MPa within TFGs during their working process. The heat-induced stresses are mainly concentrated at the position of electrodes and the contact surfaces between TFG and the packaging structure. The maximum stress in the Al electrodes is ~16 MPa, and the heat-induced stresses on the interface between the TFG and the package is ~9 MPa, which may cause significant extrusion and stretching of the interface. A large warpage exists around the edges of the PCB, the maximum displacement on the PCB is ~0.78 µm. Moreover, the large deformation of the PCB will cause a huge squeeze on the TFG, which may cause severe impacts on the TFG structures and the contact surfaces. As shown in Figure 10b, the maximum deformation between TFG and PCB may be up to 0.45 µm.

The packaging structure of this TFG consists of two main parts. One of them is the WLP realized through an anodic bonding process, which provides the necessary vacuum and protection for the sensing structure. Another one is the chip-level packaging, including wire bonding, die bonding, plastic molding, and board-level interconnection [23]. The main sources of the internal stresses are the thermomechanical stress generated by the bonded wafer and the TCE mismatch between silicon and glass.

Besides, the packaging stresses are mainly caused by the TCE mismatch between TFG and the packaging materials, and the TCE mismatch between the various materials within the TFGs is also an important source of internal stresses [24].

The simulation results show that when the TFG is working, heat-induced stresses bring about significant extrusion and stretching at the interface between the TFG and the package. Moreover, significant deformation of the TFG structure can be observed. This deformation may result in bending of the spring beams and the deformation of the proof masses, which directly leads to a non-negligible change in TFG’s inherent frequency [15]. Furthermore, the changes of the comb capacitance spacing also have significant impacts on the performance of TFG. As described in [12], the ZRO of the gyroscope is closely related to the static capacitance, gap, and inherent frequency. Besides, the stability of the frequency directly affects the accuracy of the phase-sensitive demodulation [13]. Hence, the heat-induced stresses have notable impacts on the ZRO drift of TFGs. Therefore, an efficient approach to relieve the internal and packaging stresses is in great need to reduce the impacts of Joule heat generated by TFGs on the ZRO drift, enabling gyroscopes better performances.

## 5. Temperature-Induced Stress Release Experiment

Temperature-varying cycling test is a common method adopted for failure analysis of MEMS gyroscopes, during which heat-induced positive and shear stresses between the layers within gyroscopes generate. When the high- and low-temperature cycles are applied at the interfaces, the environmental stresses bring about some cracks due to the intrinsic defects, resulting in the degradation of gyroscope performances and may eventually cause their failure. As the high- and low-temperature shocks can be applied to relieve the system stress, we conduct a temperature-induced stress release experiment by fleetly switching the high and low temperatures. The effects of internal and packaging stresses on the ZRO drift are explored by accelerating the release process of system stresses.

The four front-end PCB boards, which contain TFG, package structures, and other related parts, were placed into a temperature chamber (TL-02-70-F) for the temperature-induced stress release experiment. The minimum temperature was set as −20 °C, and the maximum temperature was 70 °C with a change rate of 10 °C/min. Besides, each peak temperature was sustained for 10 min, and a 40 cycle-temperature cycling test was conducted to sufficiently release the stresses. The duration of this experiment was about 26 h.

By cyclically applying high and low temperatures in the experiment, we introduced heat-induced stresses within the whole system. On this occasion, the residual mechanical stresses coming from wafer bonding, the interlayer stresses between SiO_2_ and Si, and the packaging stresses caused by wire bonding, molding, and mounting were dramatically released.

**Figure 11:**
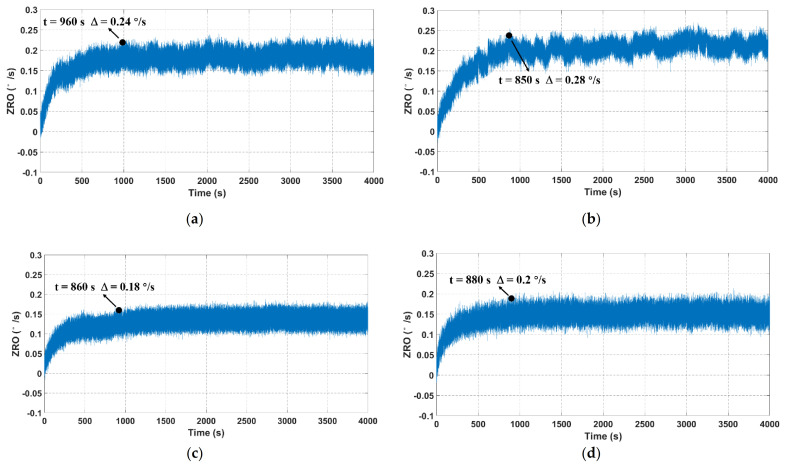
ZRO result comparisons between four stress-released TFGs. (**a**–**d**) represent the ZRO of four stress-released TFGs (samples 1–4).

To verify the effectiveness of the temperature-induced stress release experiment, ZRO measurements were conducted again for the stress-released samples, and the experimental conditions were identical as before. As depicted in Figure 11, the ZRO results also exhibited a large drift trend at the initial stage, and their variations gradually slowed down and finally stabilized. This ZRO drift tendency was approximately the same as that of as-fabricated TFGs. However, it can be observed that the duration and magnitude of ZRO drift for all of the four samples substantially reduced after the temperature-induced stress release experiment. These ZRO drift improvements are listed in Table 3. It can be seen that the ZRO drift duration was ~890 s, and its magnitude was approximately 0.23 °/s.

**Figure 12:**
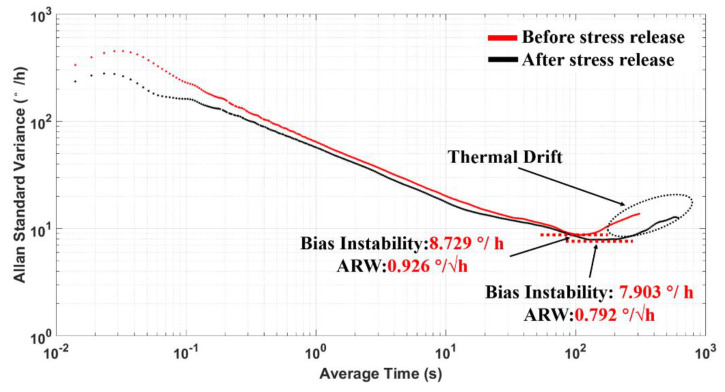
Allan standard variance plot.

To fully investigate the impacts of temperature-induced stress release experiment on TFG performances, we draw an Allan variance plot based on our collected ZRO data between untreated and stress-released samples, and the result is shown in Figure 12. It shows that the measured BI was 8.729 °/h, and the ARW reached 0.926 °/√ h before the stress release process. Nevertheless, the BI was reduced to 7.903 °/h, and the ARW was 0.792 °/√ h after stress release, indicating the effective reduction of ZRO thermal drift realized through the temperature-induced stress release experiment. Besides, no significant performance degradation was found under these experimental conditions.

By cyclically applying high and low temperatures, the sensitivity of internal and packaging stresses to the temperature variation was greatly reduced, which significantly alleviates the impacts of temperature change on device frequency characteristics during the working process of TFGs. The experimental results demonstrate that the proposed temperature-induced stress release experiment can effectively reduce the impacts of internal and packaging stresses on the ZRO drift and improve the long-term bias performances of TFGs.

## 6. Conclusions

In summary, we investigated the impacts of internal and packaging stresses of gyroscopes on their ZRO drift properties at the thermal start-up stage. Based on self-developed high-Q dual-mass TFGs, it was discovered that the gyroscope drive frequency badly drifts during its normal operation due to the heat and also the stress-induced effects. Correspondingly, the ZRO drift was non-negligible in this situation. With a finite element simulation, the inside temperature distribution together with the relationship between temperature and stresses was further revealed for our TFGs. Besides, we demonstrated a temperature-induced stress release experiment to generate thermal stresses to the TFG and packaging. By cyclically applying high and low temperatures, the temperature-related device sensitivity about stresses was restrained. Thus, the ZRO drift duration and magnitude were drastically reduced by a factor of ~1.7 and ~2.2, respectively. Moreover, a significant reduction in the thermal drift can be observed, meaning an improved long-term bias performance of TFGs. Moreover, a small low BI of 7.903 °/h, and a small ARW of 0.792 °/√ h were realized. This work provides a new thought to improve the performances of MEMS gyroscopes, bringing a new promise for their practical applications.

## Figures and Tables

**Table 1:** The duration and magnitude of ZRO drifts for four TFGs.

	Sample 1	Sample 2	Sample 3	Sample 4
f_drive_ (Hz)	11,713.5	11,856	11,800	11,515
f_sense_ (Hz)	11,612	11,757.7	11,696	11,422.6
Duration (s)	2100	2000	1900	1850
Magnitude (°/s)	0.38	0.4	0.39	0.41

**Table 2:** Dimensions and essential material parameters of components in the simulation model.

Component	Dimension (μm)	Material	TCE (1/k)	κ (W/(m · k))
Ceramic shell	280 × 280 × 140	Al_2_O_3_	6.5 × 10^−6^	35
Glass cap	200 × 200 × 100	SiO_2_	0.5 × 10^−6^	1.4
TFG structure	180 × 180 × 20	Silicon	2.5 × 10^−6^	0.21
Al electrode	15 × 15 × 22	Aluminum	23.1 × 10^−6^	237
Au electrode	20 × 20 × 30	Gold	14.2 × 10^−6^	317
Getter	50 × 50 × 10	Titanium	8.6 × 10^−6^	21.9
PCB	400 × 400 × 30	Polyimide	12.3 × 10^−6^	0.15
Conductive adhesive	220 × 220 × 10	Polyethylene	150 × 10^−6^	0.38

**Table 3:** The duration and magnitude of ZRO drift after the stress release experiment.

	Sample 1	Sample 2	Sample 3	Sample 4
Duration (s)	960	850	860	880
Improve	2.19	2.35	2.21	2.10
Magnitude (°/s)	0.24	0.28	0.18	0.2
Improve	1.58	1.43	2.17	2.05
